# Heterotopic ossification following total wrist arthroplasty: a case report

**DOI:** 10.1186/s13256-025-05258-3

**Published:** 2025-05-12

**Authors:** Sonja Verena Schmidt, Jannik Hinzmann, Maximilian Völlmecke, Christoph Wallner, Marcus Lehnhardt, Patrick Stefan Harenberg

**Affiliations:** 1 Department of Plastic Surgery and Hand Surgery, Burn Centre, Sarcoma Centre, BG University Hospital Bergmannsheil Bochum, Ruhr-University Bochum, Buerkle-de-La-Camp Platz 1, 44789 Bochum, Germany; 2 Department of Trauma Surgery, Hand and Reconstructive Surgery, Bundeswehr Central Hospital, Koblenz, Germany

**Keywords:** Total wrist arthroplasty, Heterotopic ossification, Radiocarpal arthrosis

## Abstract

**Background:**

Total wrist arthroplasty as a procedure in patients with advanced osteoarthritis has gained more popularity in recent years. As early implants had high rates of complications and newer implants have only slowly gained traction, some sequelae have not been reported yet.

**Case presentation:**

This study presents the case of a male German patient, 57 years old, with advanced osteoarthritis who received arthroplasty and presented with severely restricted range of motion 6 weeks after surgery. Radiographs revealed signs of heterotopic ossification that could be confirmed during the revision surgery. Intraoperatively, ossifications were removed and the mobile parts of the implant were changed. In the further course of the healing process, no further signs of ossifications have been reported for 1 year, but range of motion remains reduced.

**Conclusion:**

This is the first reported case of heterotopic ossification of the wrist following arthroplasty. Heterotopic ossification should be taken into consideration in cases of restricted range of motion after arthroplasty to be able to handle this complication adequately.

**Supplementary Information:**

The online version contains supplementary material available at 10.1186/s13256-025-05258-3.

## Background

Total wrist arthroplasty (TWA) has become a reliable procedure over recent decades. The first TWA dates back to 1890, when Themistocles Gluck used a ball-and-socket design made of ivory [1]. Since then, considerable progress has been made in this field. After the introduction of silicone implants and cemented implants, we have been led to the development of cementless implants for modern total wrist arthroplasty, as performed regularly these days [2]. Over the last two decades, indications besides rheumatoid arthritis have increased steadily. Patients with posttraumatic or primary osteoarthrosis tend to be younger and more active. Therefore, total wrist arthroplasty must be a reliable and long-lasting option. Patients report pain relief after surgery, and although complication rates are similar compared with arthrodesis, Disabilities of the Arm, Shoulder, and Hand scores are significantly lower in arthroplasties [3]. Nevertheless, relatively little is known about the long-term outcomes of wrist arthroplasty as it is still evolving. Therefore, documentation of complications and continuous research in the field of wrist arthroplasty remain important to improve this relevant procedure further. 

Heterotopic ossification (HO) is a complex pathologic process that can occur owing to different circumstances. It is defined as extraskeletal bone tissue formation in muscle and soft tissue and is associated with abnormal tissue repair [4].

HO includes a broad spectrum and most commonly develops after trauma, major orthopedic surgeries such as hip arthroplasty, or thermal and neurologic injuries [5]. One of the most common theories assumes that, owing to stress in the local tissue environment because of the different forms of trauma, various circumstances such as oxygen alterations, pH, availability of micronutrients, or mechanical stimuli have an impact on cell-mediated interactions. This eventually leads to conversion of progenitor cells to osteogenic precursor cells, which finally leads to the development of heterotopic ossification in these areas [5]. Regarding this theory, the local stress to the affected area seems to be the trigger. However, distant regions can also develop HO, especially after traumas such as neurologic impairment or burns. Different factors play important roles in this mechanism. Burns, for example, upregulate the bone morphogenetic proteins (BMP)-2/4 pathway, which is highly relevant for osteoinductive properties. Furthermore, ischemic conditions induce hypoxia-inducible factor-1 alpha (HIF-1α), which affects different cytokines that are responsible for endothelial cell mobility, recruitment, and proliferation [5]. These mechanisms indicate that HO is based on a highly complex pathologic mechanism. 

As heterotopic ossification is a relevant functional complication, different approaches for prophylaxis, such as non-steroidal anti-inflammatory drugs (NSAIDs), bisphosphonate, or radiation, have been the object of various research endeavors. Moreover, treatment and pathologic processes are also objects of current research. In orthopedic surgery, HO remains an extremely relevant complication after arthoplasties that should be taken into consideration in intricate cases. 

## Case presentation

We present a male, white German patient, 57 years old, who has had a severe motorcycle accident in 2016 in which he suffered a transscaphoid perilunate fracture dislocation with simultaneous fracture of the capitate and the triquetrum, and a multifragmentary fracture of the styloid process of the radius. Initially, the injury had been treated with repositioning and establishment of external fixation of the wrist. The following day, a carpal tunnel release, division of the first extensor tendon sheath, and open reduction internal fixation (ORIF) of the capitatum and scaphoid using two Herbert screws (2.4 × 20 and 22 mm) as well as fixation of the styloid process of the radius with two 1.25-mm K-wires had been performed. After 2 weeks, the K-wires were replaced with a T-plate and a split-thickness skin graft was transplanted to the volar side of the wrist. Hardware removal was performed after another 6 weeks. One year after the trauma, the patient reported sufficient coping with daily life with a remaining range of motion (ROM) of 30–0–10° (extension/flexion). X-ray already showed signs of radiocarpal arthrosis (Supplementary Fig. 1). 

**Fig. 1:**
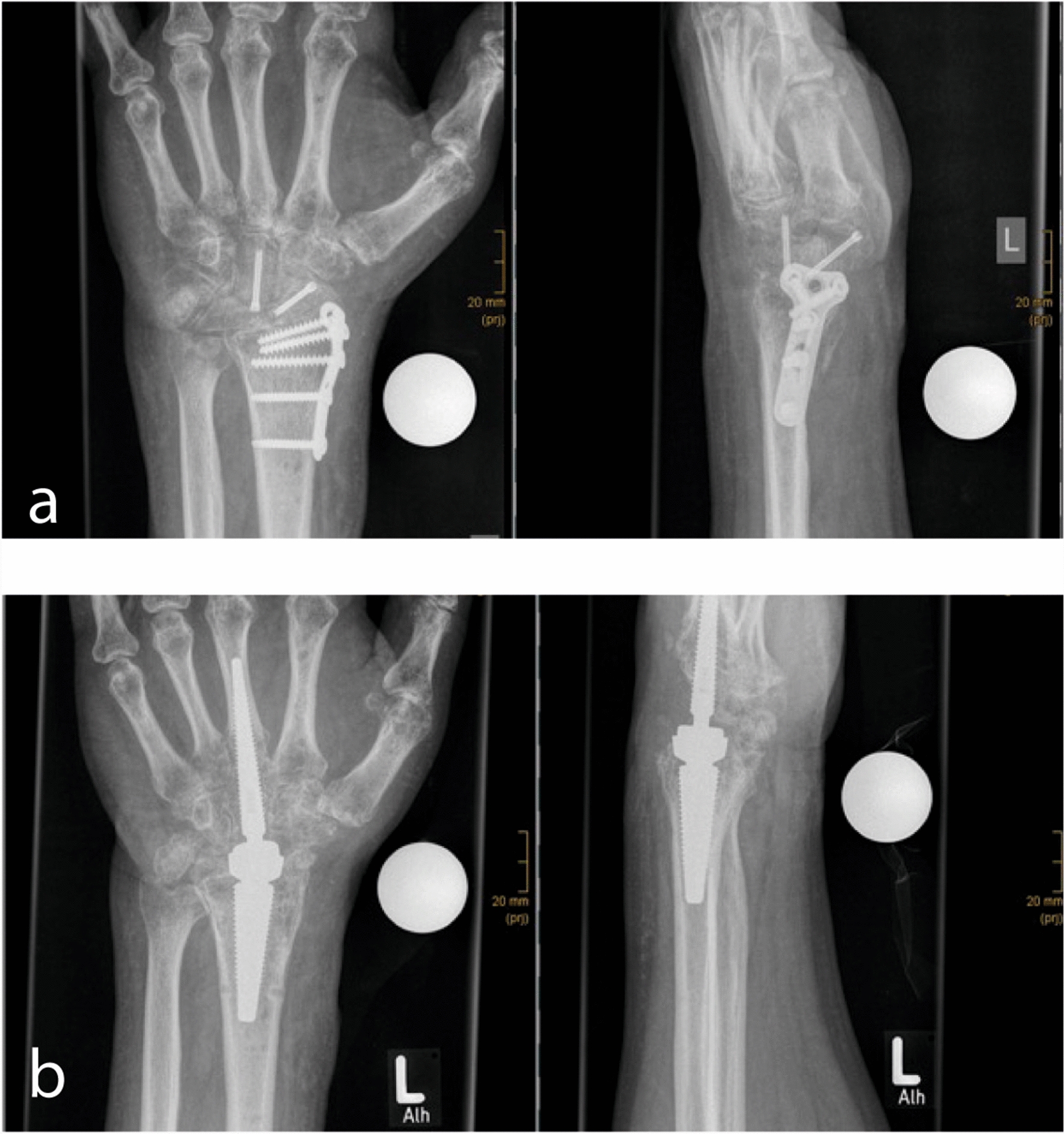
X-rays of the left hand of the patient in the anterior–posterior and lateral views. **a** Preoperative x-ray. Herbert screws and T-plate of the initial surgeries are still in situ. Advanced mediocarpal and radiocarpal arthrosis of the left wrist. **b** Preoperative X-ray with sign of heterotopic ossification in the joint gap

Seven years after the trauma, the patient presented again with increasing pain and progressive restriction of ROM of the left wrist (Fig. 1a). Computed tomography showed signs of advanced mediocarpal and radiocarpal arthrosis. The patient, who works as a steelworker, was very eager to keep as much ROM in his wrist as possible. Therefore, a total wrist arthroplasty was recommended. In our department, we perform TWAs with the MOTEC-prosthesis, a ball-and-socket design that allows up to 80° theoretical range of motion (ROM) in all directions. The metacarpal head is made of cobalt-chromium-molybdenum (CoCrMo) alloy, and the radius cup is made of carbon-fiber-reinforced polyetheretherketone (CFR-PEEK) [6]. The patient’s surgery was performed without any complications (Supplementary Fig. 2). The hospital stay was 4 days, without occurrence of any issues. Postoperatively, the wrist was immobilized for 6 weeks in an individualized long thumb orthosis.

**Fig. 2:**
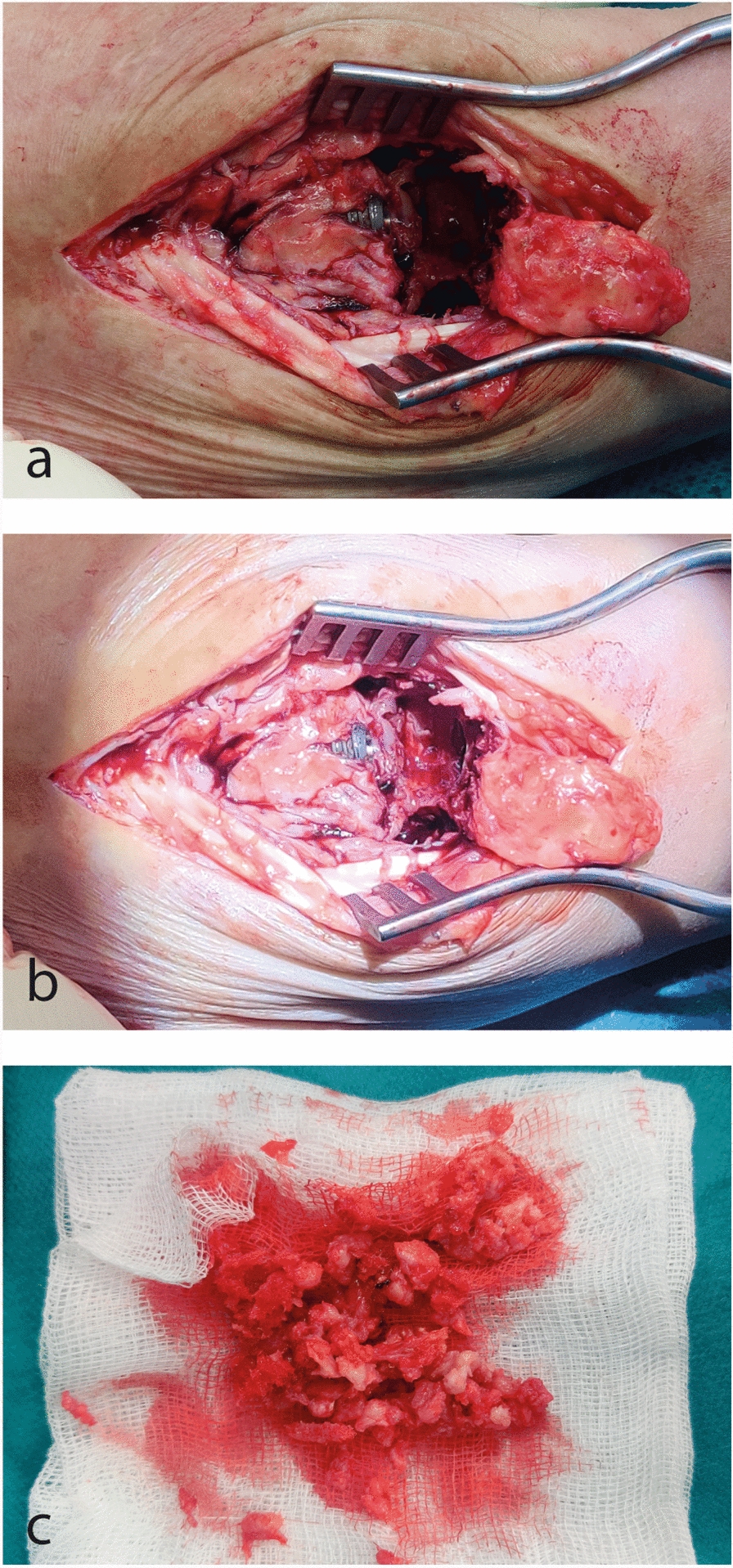
Intraoperative clinical images. **a**, **b** Images of the situs showing bone-like tissue around the mobile parts of the implant. **c** Resected bony tissue

**Fig. 3:**
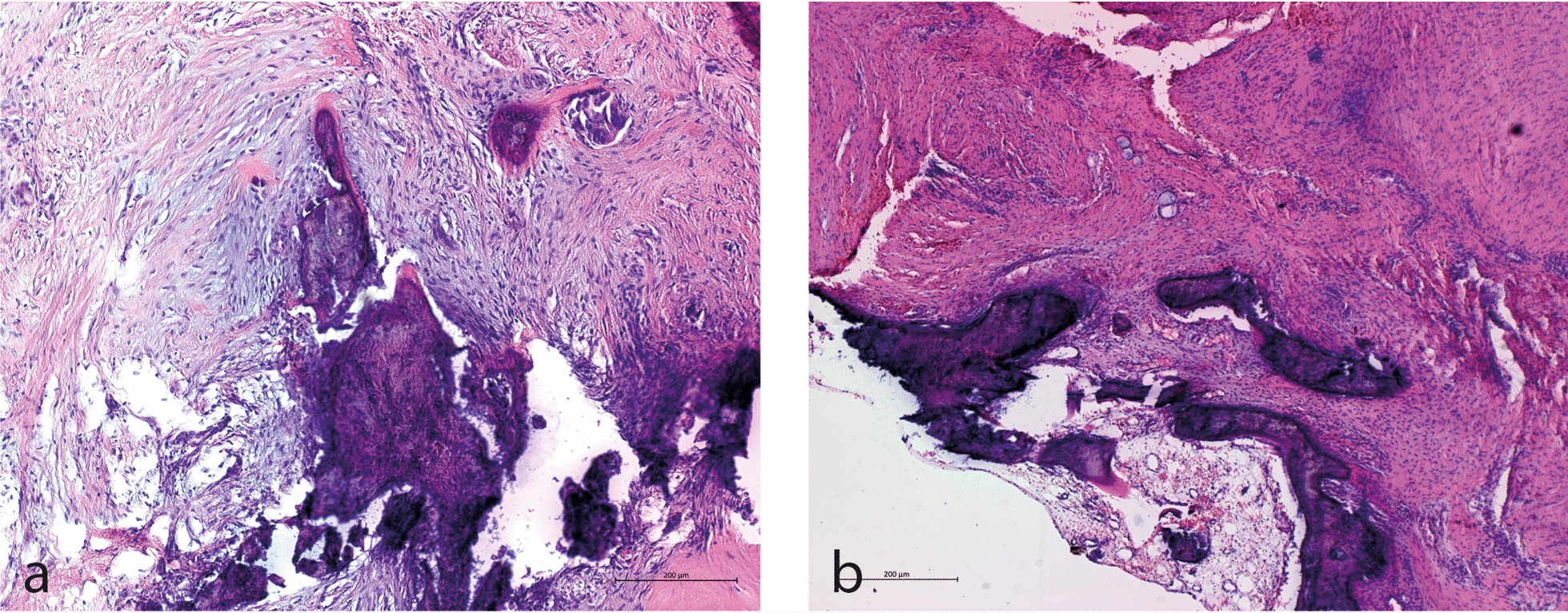
Histopathological images of the resected tissue. **a** Extensively fibrosed connective tissue with regressive structural changes. Prominent sclerosed bone fragments are embedded within the fibrous stroma, accompanied by a chronic granulating inflammatory response. **b** Dense fibrotic connective tissue interspersed with irregular, necrotic bone fragments. A surrounding chronic inflammatory infiltrate and signs of ongoing remodeling are visible, indicating an active yet disorganized repair process

At 6 weeks after the implantation, a routine X-ray of the wrist together with a clinical examination was performed. The patient complained about severely restricted ROM as well as pain, and the X-ray revealed excessive ossification in the joint gap, especially at the volar aspect of the wrist (Fig. 1b). Because of the high level of suspicion for heterotopic ossification, revision surgery was planned. In the procedure, mobile parts of the implant were removed and the ossifications, which were located around the implant but especially volarly, were resected carefully with a rongeur and sent to pathology. After resection of all heterotopic ossifications, mobile parts of the prosthesis and the joint were rinsed with iodine solution and the mobile parts were placed back (Fig. 2). Intraoperatively, examination of the wrist presented sufficient ROM. Postoperative procedure implicated immobilization for only 2 weeks and rapid physical exercise afterwards. Furthermore, ossification prophylaxis with high-dose NSAID (ibuprofen 800 mg, 3/day) was administered for 6 weeks. Histopathological processing of the ossifications revealed regressively changed and fibrosed connective tissue with a chronic granulating inflammatory reaction with sclerosed bone fragments. These results fit the clinical findings of heterotopic ossification (Fig. 3).

**Fig. 4:**
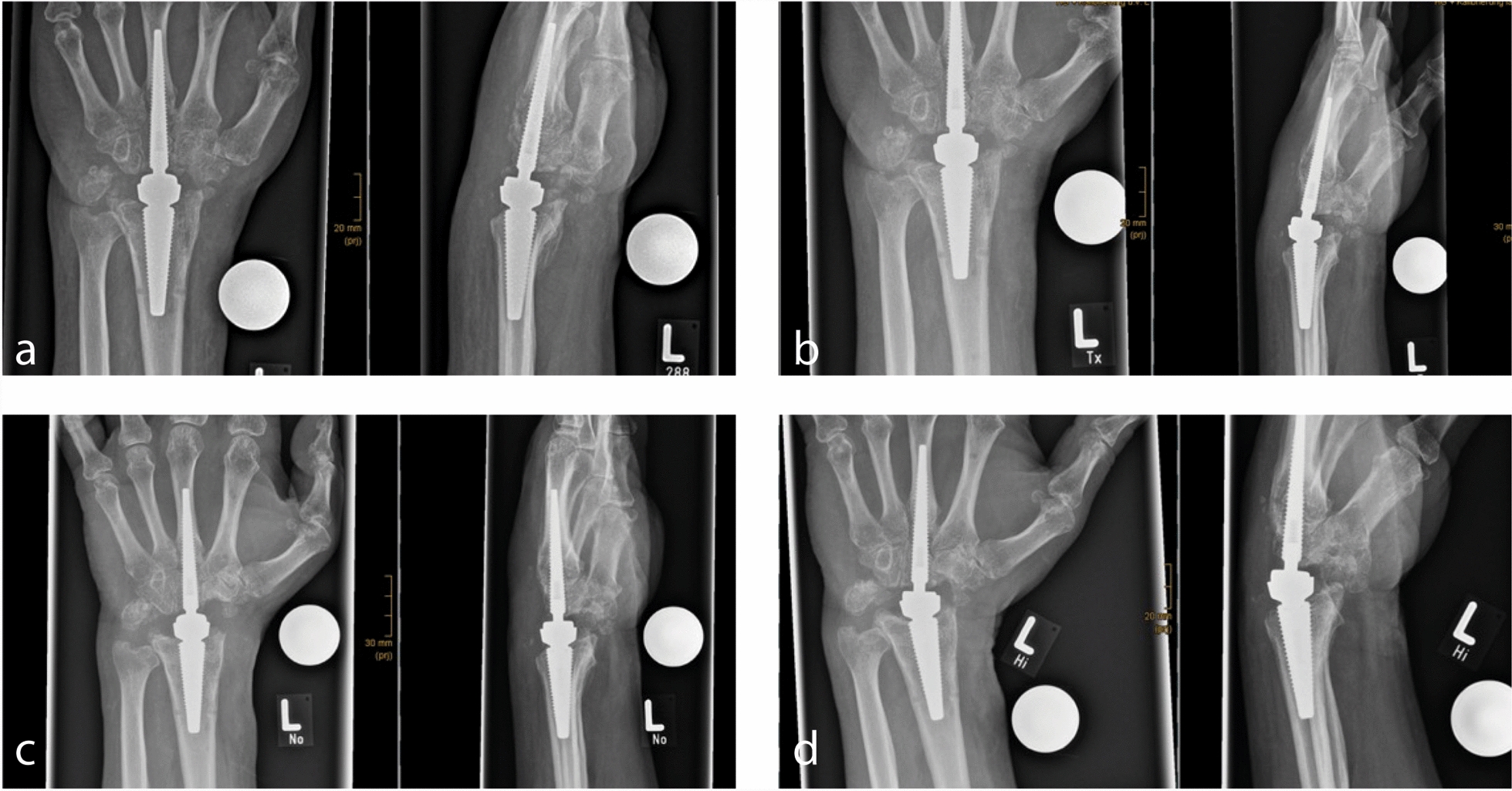
X-rays of the left hand as part of aftercare examination. **a** X-rays in anterior–posterior and lateral views, 6 weeks after revision surgery. **b** X-rays in anterior–posterior and lateral views, 3 month after revision surgery. **a**, **c** X-rays in anterior–posterior and lateral views, 6 months after revision surgery. **d** X-rays in anterior–posterior and lateral views, 1 year after revision surgery

Follow-up was carried out at 2 months, 4 months, and 8 months after revision surgery. ROM improved drastically in all directions during the postoperative course, and the patient only reported pain when doing maximal movements. The patient still reports feelings of pressure within the joint and remaining dysesthesia in the skin territory of the superficial branch of the radial nerve. Follow-up X-rays showed no further signs of HO (Fig. 4). The patient is very satisfied with the result and could continue his job as a steelworker (Table 1).

**Table 1:** Range of motion of the wrist in all three planes by neutral zero method: extension/flexion, radial and ulnar deviation, pronation/supination in degrees as well as mean maximal dynamic grip force in kilograms

	ROM preoperatively		ROM 6 weeks postoperative		ROM 8 weeks after revision		ROM 16 weeks after revision		ROM 10 months after revision	
	Right side	Left side	Right side	Left side	Right side	Left side	Right side	Left side	Right side	Left side
Extension/flexion (°)	70–0–70°	25–0–15°	70–0–75°	0–0–30°	60–0–70°	25–0–40°	75–0–75°	40–0–45°	60–0–75°	65–0–80°
Radial/ulnar deviation (°)	25–0–35°	0–0–5°	30–0–30°	0–0–10°	20–0–30°	10–0–15°	25–0–30°	15–0–30°	15–0–40°	10–0–30°
Pronation/supination (°)	90–0–90°	75–0–75°	90–0–90°	90–0–45°	90–0–90°	75–0–90°	90–0–90°	90–0–75°	80–0–80°	65–0–80°
Mean force (kg)	38 kg	5 kg	47 kg	3.67 kg	46 kg	4.67 kg	49.33 kg	10 kg	44.33 kg	7 kg

ROM of affected (left) and unaffected (right) wrist are compared. For maximal force, the average value of three different measurements is listed

## Discussion and conclusion

We present herein the first documented occurrence of heterotopic ossification after TWA. HO can develop owing to different reasons such as neurologic injuries, post trauma, or after orthopedic surgeries. In cases of hip endoprosthesis, for example, HO is a frequently described postoperative complication that is object of different research projects. Concerning the occurrence of HO after hip endoprosthesis, multiple risk factors have been described, such as male sex, usage of cemented prosthesis, bilateral arthroplasty, previous history of HO, or ankylosing spondylitis [7]. It remains unclear whether these risk factors are equally relevant for wrist arthroplasty. Factors such as the surgical approach, implant type, and intraoperative trauma may also play a more critical role in smaller joints, warranting further investigation.

In some works, serum markers are discussed as predictive values for the development of HO. Alkaline phosphatase or C telopeptide of type I collagen (CTX-1) are discussed in different papers but are still not predictive enough to assess routine measurements postoperatively [7]. In the case of wrist arthroplasty, the usage of bone metabolic turnover markers seems to be even less useful as the amount of ossification in relation to the ossification after hip arthroplasty is quite small. Thus, routine assessment of the development of HO after wrist arthroplasty may be discussed on the basis of X-rays of the wrist taken at certain time intervals postoperatively but without screening for serum markers. Also, in our case, the heterotopic ossification was detected via X-rays and matched the clinical impression of restricted mobility. Therefore, further work should focus on postoperative protocols for certain X-ray intervals to standardize routine postoperative imaging.

For HO of the hip, there is a classification to distinguish different degrees of severity. The Brooker classification consists of four grades with grade III and IV being clinically relevant. Furthermore, there is the Classification of Hastings and Graham for HO of the elbow, in which radiographic evidence of ossification without functional deficit is differentiated from forms with limitation in different movement axes [8]. This classification seems reasonable for HO of the forearm and also the wrist as it is more accurate concerning the remaining mobility in small joints as elbow and wrist and therefore applicable for our case, which would be grade IIB regarding the Classification of Hastings and Graham.

In conclusion, HO is a relevant complication in surgery to which several research endeavors have been dedicated. It has not previously been reported in the context of total wrist arthroplasty, thus this case marks a relevant observation. After TWA, regular X-rays, especially when detecting restricted ROM, should be performed to screen for heterotopic ossifications. In cases of restricted ROM, revision surgery as well as prophylactic treatment with NSAIDs should be considered to treat this rare complication.

## Supplementary Information

Supplementary Material 1. Fig. 1. X-rays of the left hand of the patient in the a.p. and lateral view. (a) Day of the accident, the x-ray shows a transscaphoid perilunate fracture dislocation with simultaneous fracture of the capitatum, triquetrum and a multifragmentary fractur of the styloid process of the radius. (b) First day after the accident and the two initial surgeries (establishment of external fixation, ORIF of capitatum and scaphoid using two Herbert screws (2.4 × 20, and 22 mm) and fixation of styloid process using two 1.25 mm K-wires. (c) Six weeks after the accident. K-wires at the styloid process have been replaced with T-plate. (d) Eight weeks after the accident. Removal of remaining transfixation and external fixation has been performed.

Supplementary Material 2. Fig. 2. Intraoperative x-rays after initial implantation of the MOTEC prosthesis. (a) a.p. view of the implanted wrist arthroplasty. (b) lateral view of TWA.

## Data Availability

Further supporting data can be obtained by contacting the corresponding author.

## References

[1] Ritt MJPF, Stuart PR, Naggar L, *et al*. The early history of arthroplasty of the wrist. From amputation to total wrist implant. J Hand Surg Br. 1994;19:778–82. 7706886 10.1016/0266-7681(94)90257-7

[2] Halim A, Weiss APC. Total wrist arthroplasty. J Hand Surg Am. 2017;42:198–209. 28111060 10.1016/j.jhsa.2016.12.004

[3] Nydick JA, Watt JF, Garcia MJ, *et al*. Clinical outcomes of arthrodesis and arthroplasty for the treatment of posttraumatic wrist arthritis. J Hand Surg Am. 2013;38:899–903. 23561729 10.1016/j.jhsa.2013.02.013

[4] Meyers C, Lisiecki J, Miller S, *et al*. Heterotopic ossification: a comprehensive review. JBMR Plus. 2019. 10.1002/JBM4.10172. (**Epub ahead of print 1 April 2019**). 31044187 10.1002/jbm4.10172 PMC6478587

[5] Ranganathan K, Loder S, Agarwal S, *et al*. Heterotopic ossification: basic-science principles and clinical correlates. J Bone Joint Surg Am. 2015;97:1101. 26135077 10.2106/JBJS.N.01056 PMC6948799

[6] Motec Wrist Joint Prosthesis—Product brochure—P125-28-1-20231219.pdf—Google Drive, https://drive.google.com/file/d/1alBzPI-mLPQw87oQlPwSves8OAE4QROL/view. Accessed 15 July 2024.

[7] Łȩgosz P, Otworowski M, Sibilska A, *et al*. Heterotopic ossification: a challenging complication of total hip arthroplasty: risk factors, diagnosis, prophylaxis, and treatment. Biomed Res Int. 2019. 10.1155/2019/3860142. (**Epub ahead of print 2019**). 31119167 10.1155/2019/3860142 PMC6500709

[8] Hirakawa A, Komura S, Kuramitsu N, *et al*. The incidence of heterotopic ossification in surgically and non-surgically treated elbow fractures at a municipal hospital in Japan. J Hand Surg Asian Pac. 2023;28:472–8. 10.1142/S2424835523500546 37758493

