# Correction: Heterotopic ossification following total wrist arthroplasty: a case report

**DOI:** 10.1186/s13256-025-05487-6

**Published:** 2025-08-19

**Authors:** Sonja Verena Schmidt, Jannik Hinzmann, Maximilian Völlmecke, Christoph Wallner, Marcus Lehnhardt, Patrick Stefan Harenberg

**Affiliations:** 1 Department of Plastic Surgery and Hand Surgery, Burn Centre, Sarcoma Centre, BG University Hospital Bergmannsheil Bochum, Ruhr-University Bochum, Buerkle‑de‑La‑Camp Platz 1, 44789 Bochum, Germany; 2 Department of Trauma Surgery, Hand and Reconstructive Surgery, Bundeswehr Central Hospital, Koblenz, Germany

**Correction** **:** **Journal of Medical Case Reports (2025) 19:215** 10.1186/s13256-025-05258-3

Following publication of the original article [1], the authors identified an error in the author name of Patrick Stefan Harenberg.

The incorrect author name is: Patrick Simon Harenberg

The correct author name is: Patrick Stefan Harenberg

The author group has been updated above and the original article [1] has been corrected.
