# Supplementary figures and images for: Heterotopic ossification following total wrist arthroplasty: a case report
Source: J Med Case Rep. 2025 May 12;19:215. doi: 10.1186/s13256-025-05258-3 (PMC12067731; doi:10.1186/s13256-025-05258-3)

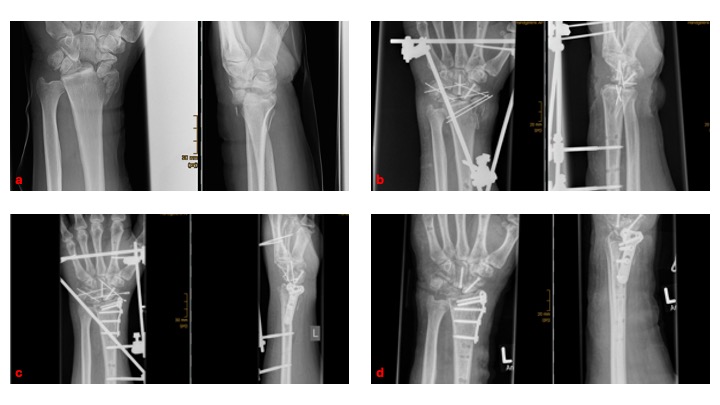

Supplement: Supplementary file 1 — Supplementary Material 1. Fig. 1. X-rays of the left hand of the patient in the a.p. and lateral view. (a) Day of the accident, the x-ray shows a transscaphoid perilunate fracture dislocation with simultaneous fracture of the capitatum, triquetrum and a multifragmentary fractur of the styloid process of the radius. (b) First day after the accident and the two initial surgeries (establishment of external fixation, ORIF of capitatum and scaphoid using two Herbert screws (2.4 × 20, and 22 mm) and fixation of styloid process using two 1.25 mm K-wires. (c) Six weeks after the accident. K-wires at the styloid process have been replaced with T-plate. (d) Eight weeks after the accident. Removal of remaining transfixation and external fixation has been performed. [file 13256_2025_5258_MOESM1_ESM.jpg]

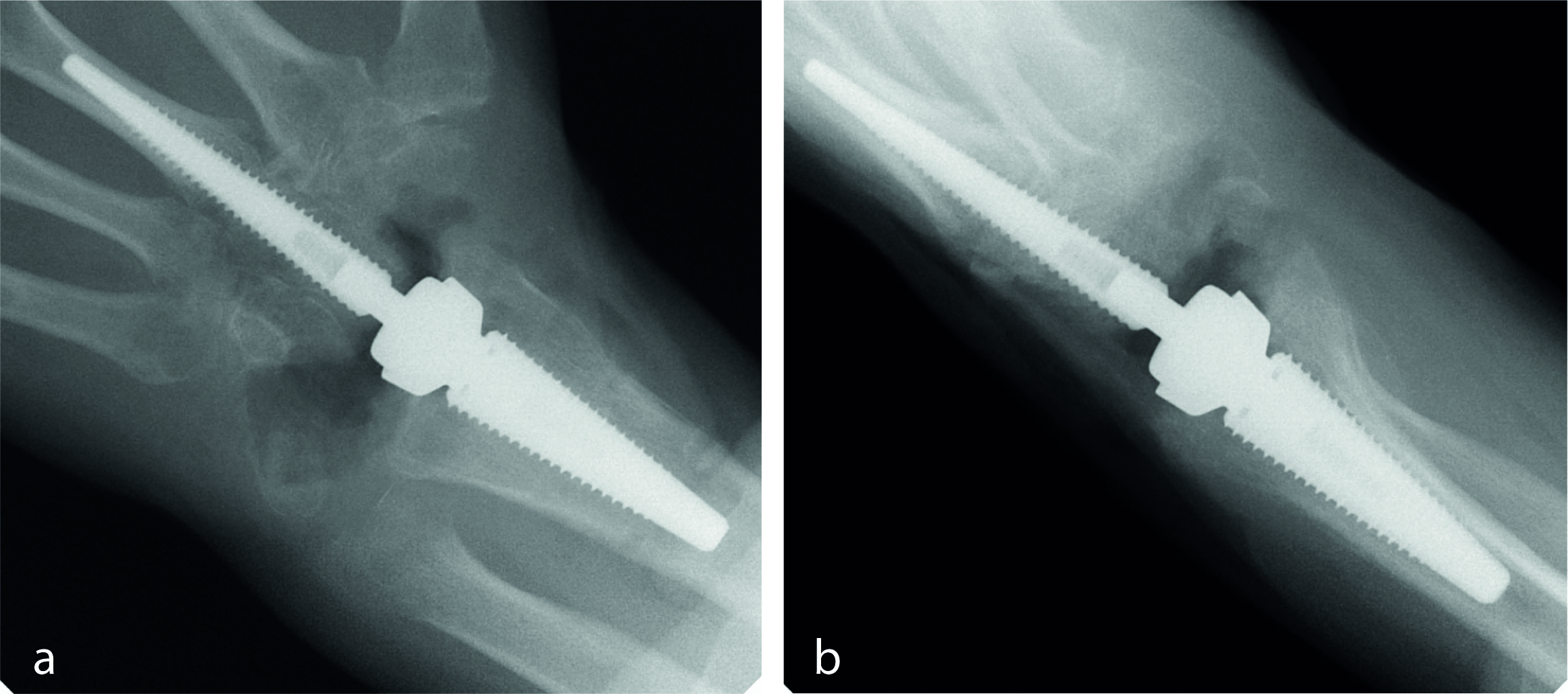

Supplement: Supplementary file 2 — Supplementary Material 2. Fig. 2. Intraoperative x-rays after initial implantation of the MOTEC prosthesis. (a) a.p. view of the implanted wrist arthroplasty. (b) lateral view of TWA. [file 13256_2025_5258_MOESM2_ESM.jpg]
